# Predicting progression to active tuberculosis: A rate-limiting step on the path to elimination

**DOI:** 10.1371/journal.pmed.1002814

**Published:** 2019-05-24

**Authors:** Ajit Lalvani, Luis C. Berrocal-Almanza, Alice Halliday

**Affiliations:** 1 National Institute for Health Research Health Protection Research Unit in Respiratory Infections, Imperial College London, London, United Kingdom; 2 Tuberculosis Research Centre, National Heart and Lung Institute, St Mary's Campus, Imperial College London, London, United Kingdom; 3 Cellular and Molecular Medicine, University of Bristol, Bristol, United Kingdom

## Abstract

In a Perspective, Ajit Lalvani and colleagues discuss new approaches to predicting progression to active tuberculosis.

## Current tools are not fit for purpose to deliver tuberculosis elimination

Tuberculosis (TB) is the leading cause of mortality from an infectious disease worldwide, with 1.3 million deaths in 2017 [1]. Once TB infection has progressed to active pulmonary TB, onward transmission is common, propagating the pandemic. The detection and preventive treatment of persons with latent infection before the development of active, infectious TB is the cornerstone of TB control in low-incidence regions and is essential for achieving elimination.

However, the current state-of-the-art tools for diagnosing latent infection, the interferon-γ release assays (IGRAs), predict very poorly who will progress to active TB. The largest study to evaluate IGRAs and tuberculin skin tests (TSTs) recently showed that in a low-incidence setting among TB contacts and recent immigrants from high-incidence countries, although the negative predictive values (NPVs) of IGRAs and TSTs are >99%, positive predictive values (PPVs) are only 3%–4% [2]. Thus, whilst a negative IGRA result reliably excludes progression to TB, only 3%–4% of those with positive results progress; in other words, the number of TB-exposed IGRA-positive persons that need to be treated (NNT) to prevent a single case of TB is 25–33, assuming 100% adherence to and effectiveness of preventive therapy. Considering the toxicity and cost of preventive treatment, TB elimination will not be possible with TST or IGRA, even if shorter preventive regimes become available soon [3,4].

A major breakthrough would be a diagnostic tool able to accurately predict which individuals will progress to active disease after infection. A World Health Organization (WHO) consensus statement identified an optimal target product profile that should predict risk of progression to TB within two years after infection with sensitivity and specificity ≥90% or at least ≥75%, a high PPV, and a low NNT [5]. Indeed, it is estimated that a large proportion of the 10 million new cases reported in 2017 [1] could have been averted through preventive treatment [6] with such a test.

## More sophisticated measures of host responses can identify incipient preclinical TB before development of symptoms

A series of studies in the last few years described the potential of genome-wide gene-expression (transcriptomic) signatures from whole blood to differentiate symptomatic active TB from latent TB infection and other diseases; however, determining the clinical usefulness of such RNA signatures will require a prospective study of patients with suspected active TB in routine clinical practice. Notwithstanding, investigators from the South African Tuberculosis Vaccine Initiative (SATVI) applied this technology to address a different clinical need: is there an RNA signature that could identify progression of latent TB infection? In a large, well-designed, prospective cohort study, the investigators identified a 16-gene blood RNA signature that appears to predict progression to active disease [7]. This new correlate of risk had 66.1% sensitivity and 80.6% specificity within 12 months prior to diagnosis. Of note, the signature’s sensitivity increased nearer to the time of diagnosis, being 71.2% within 6 months before diagnosis [7]. Although it has been questioned whether this RNA risk signature would represent a significant improvement in practice over IGRAs because of its low specificity [8], it is an important discovery that warrants further investigation and validation. The observation that the accuracy of the risk signature increased as the time of diagnosis approached suggests that it detects incipient preclinical disease in asymptomatic individuals. Whilst it may not therefore be a true predictor of progression from latent infection to active TB, it could nonetheless have a great impact in TB control because population-wide identification and treatment of these persons could substantially reduce transmission. Very recently, new risk signatures with only 4 and 3 genes, respectively, showing apparently similar performance have been reported [9,10].

Proving the clinical and public health benefits of this signature would require demonstrating whether it can detect disease progression in the general population in a high-incidence setting [11]. In addition, it is not clear whether risk signature-positive persons would benefit from preventive treatment or how the signature would perform in a head-to-head comparison with IGRAs. Some of these key questions are being addressed in the Correlate of Risk Targeted Intervention Study (CORTIS) trial, which aims to provide proof-of-concept for a risk-signature–targeted test-and-treat strategy by randomizing those with a positive risk signature to preventive treatment or clinical follow-up and comparing the subsequent incidence of TB in the two groups [11]. Finally, although these RNA signatures have been validated in several African populations independent of the South African adolescent cohort in which they were originally derived, they have not yet been assessed in a population in a low-incidence setting.

## The importance of recent infection for targeting control efforts and predicting progression to active TB

Recent infection is an extremely important risk factor for progression to active TB, and, although TB also results from remotely acquired infections, a substantial proportion of cases globally arise from recently acquired infections [12]. Targeting latent infection at risk of progression therefore requires identifying and treating those with recently acquired infections, a task that is complicated by the high probability of reinfection in high-incidence countries [12]. The high NPV of IGRAs is a powerful tool for TB control and could be harnessed in a two-step strategy if we could risk-stratify IGRA-positive persons to decrease the NNT for preventing a case of TB by, for example, identifying and treating only those whose infection was recently acquired. Such an approach would capitalise on the high NPV of IGRA whilst refining its low PPV by using a second test to risk-stratify. In this regard, immunological differences between recently acquired and remotely acquired infections are beginning to emerge [13–15], including a population of antigen-specific effector T cells that correlates with recent infection [13] and therefore potentially with risk of progression to active TB.

## Concluding remarks

We are at an exciting moment in the evolution of our TB prevention toolkit. Recently identified host-response signatures are beginning to turn the tide on the once seemingly intractable challenge of accurately predicting progression to active TB. Test-and-treat strategies based on new tests will be influenced by the epidemiological context, the incidence of TB, whether the new technologies are point-of-care or laboratory-based, and whether the new tests are applied to IGRA-positive persons as part of a two-step approach (requiring a test with high PPV) or to people not previously screened by IGRA (requiring a test with high NPV and high PPV). Ultimately, evaluations should be performed in routine practice in the relevant target population, including relevant outcomes for patients and health systems and setting-specific cost-effectiveness analyses. However, as with all new technological advances in our growing TB toolkit, no test will be enough when confronted with weak healthcare systems that fail the poorest in society and living conditions that fuel the spread of this disease.

## Abbreviations

CORTIS: Correlate of Risk Targeted Intervention Study
IGRA: interferon-γ release assay
NNT: number needed to treat
NPV: negative predictive value
PPV: positive predictive value
SATVI: South African Tuberculosis Vaccine Initiative
TB: tuberculosis
TST: tuberculin skin test
WHO: World Health Organization
